# Elements in nucleotide sensing and hydrolysis of the AAA+ disaggregation machine ClpB: a structure-based mechanistic dissection of a molecular motor

**DOI:** 10.1107/S1399004713030629

**Published:** 2014-01-31

**Authors:** Cathleen Zeymer, Thomas R. M. Barends, Nicolas D. Werbeck, Ilme Schlichting, Jochen Reinstein

**Affiliations:** a Department of Biomolecular Mechanisms, Max Planck Institute for Medical Research, Jahnstrasse 29, 69120 Heidelberg, Germany

**Keywords:** ClpB, AAA+ protein, molecular chaperones, molecular motors, transient kinetics, enzyme mechanisms, nucleotide sensing

## Abstract

ATPases of the AAA+ superfamily are large oligomeric molecular machines that remodel their substrates by converting the energy from ATP hydrolysis into mechanical force. This study focuses on the molecular chaperone ClpB, the bacterial homologue of Hsp104, which reactivates aggregated proteins under cellular stress conditions. Based on high-resolution crystal structures in different nucleotide states, mutational analysis and nucleotide-binding kinetics experiments, the ATPase cycle of the C-terminal nucleotide-binding domain (NBD2), one of the motor subunits of this AAA+ disaggregation machine, is dissected mechanistically. The results provide insights into nucleotide sensing, explaining how the conserved sensor 2 motif contributes to the discrimination between ADP and ATP binding. Furthermore, the role of a conserved active-site arginine (Arg621), which controls binding of the essential Mg^2+^ ion, is described. Finally, a hypothesis is presented as to how the ATPase activity is regulated by a conformational switch that involves the essential Walker A lysine. In the proposed model, an unusual side-chain conformation of this highly conserved residue stabilizes a catalytically inactive state, thereby avoiding unnecessary ATP hydrolysis.

## Abstract 2

High-resolution crystal structures together with mutational analysis and transient kinetics experiments were utilized to understand nucleotide sensing and the regulation of the ATPase cycle in an AAA+ molecular motor.

## 1. Introduction   

The molecular chaperone ClpB, the bacterial homologue of Hsp104, is a disaggregation machine that provides thermotolerance by resolubilizing and reactivating aggregated proteins in concert with the DnaK (Hsp70) chaperone system (Sanchez & Lindquist, 1990 ▶; Doyle & Wickner, 2009 ▶). ClpB belongs to the family of AAA+ proteins (ATPases associated with various cellular activities) that comprises a wide range of molecular machines which utilize the energy from ATP hydrolysis to remodel their respective substrates (Tucker & Sallai, 2007 ▶; Hanson & Whiteheart, 2005 ▶). Like other members of the AAA+ superfamily, ClpB forms hexameric ring structures (Lee *et al.*, 2007 ▶). The disaggregation process is highly complex and involves the translocation of polypeptide chains through the central pore of the hexameric assembly (Weibezahn *et al.*, 2004 ▶), which is achieved by highly coordinated action of the different ATPase sites within the complex (Werbeck *et al.*, 2008 ▶; Franzmann *et al.*, 2011 ▶). Recent studies have revealed mechanistic details regarding the interplay between ClpB and its co-chaperone DnaK (Seyffer *et al.*, 2012 ▶; Rosenzweig *et al.*, 2013 ▶), which is believed to recruit ClpB to the aggregates (Winkler *et al.*, 2012 ▶).

**Figure 1:**
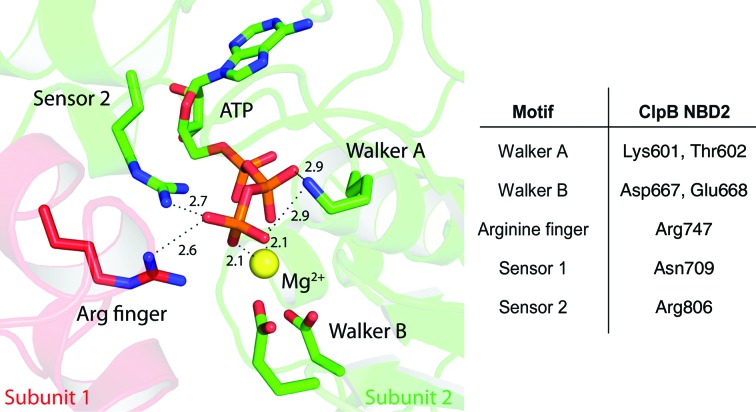
Model of a typical AAA+ catalytic site in its active conformation. The catalytic site is located at the interface between two neighbouring subunits (red and green) in the oligomeric AAA+ complex. The Walker A lysine, which is crucial for nucleotide binding and catalysis, interacts directly with the phosphates of the bound nucleotide. The essential Mg^2+^ ion (yellow) is bound between the β- and γ-­phosphates of ATP. The Walker B aspartate coordinates the Mg^2+^ ion (either directly or *via* the water molecules of the hydration shell). The Walker B glutamate is positioned such that it can activate the attacking water molecule. A conserved catalytically essential arginine residue from the neighbouring subunit (arginine finger) contacts the bound nucleotide. Conserved sensor-motif residues are in close proximity and are involved in nucleotide-state sensing and allosteric communication. This figure was generated based on the structure of the *E. coli* clamp loader (PDB entry 3glf; Simonetta *et al.*, 2009 ▶). Distances are given in Å. Corresponding residues in ClpB NBD2 are listed next to the model.

The nucleotide-binding domains (NBDs) of AAA+ proteins constitute the motor subunits of the molecular machine. They share highly conserved structural features (Wendler *et al.*, 2012 ▶). The overall fold of an NBD consists of an α/β core domain, which possesses a central, five-stranded, parallel β-sheet surrounded by five α-helices, and a C-terminal helical bundle. A unifying feature that is shared by all AAA+ proteins, but is also more generally found amongst various families of ATPases and GTPases, is the phosphate-binding loop (P-loop) or Walker A motif characterized by the consensus sequence G*XXXX*GKT/S (Walker *et al.*, 1982 ▶; Saraste *et al.*, 1990 ▶). The P-loop residues form interactions with the phosphates of the bound nucleotide. In particular, the highly conserved Walker A lysine was found to be absolutely essential for nucleotide binding and catalysis. The second common feature in AAA+ proteins is the Walker B motif *hhhh*DE, where *h* is a hydrophobic amino acid (Walker *et al.*, 1982 ▶). The highly conserved Walker B glutamate is believed to activate the attacking water molecule during ATP hydrolysis. AAA+ proteins are active as oligomers, with the catalytic site being located at the interface between two NBD subunits in the oligomer. Highly conserved arginine residues, termed arginine fingers, are located in this interface and are believed to contact the nucleotide of the neighbouring subunit (Ogura *et al.*, 2004 ▶). Furthermore, conserved sensor motifs have been identified and proposed to be involved in nucleotide-state sensing, allosteric communication or discrimination between the ADP and ATP states, which is a prerequisite for a functioning ATPase motor (Guenther *et al.*, 1997 ▶). The sensor 1 motif contains a conserved polar residue (N/T) that is essential for ATP hydrolysis. The sensor 2 motif (GAR) located on the C-terminal helical bundle contains a conserved arginine residue that can interact directly with the nucleotide. A model of the typical AAA+ catalytic site with the essential residues in their active conformation is depicted in Fig. 1 ▶.

The AAA+ disaggregation machines Hsp104 and ClpB possess two NBDs per monomer, thus forming double-ring structures with 12 potential ATPase sites in the active hexameric complex. In order to dissect interfering processes in the oligomeric context of the full-length protein, the N-­terminal nucleotide-binding domain (NBD1) and the C-­terminal nucleotide-binding domain (NBD2) of ClpB from *Thermus thermophilus* were studied as separately expressed constructs with regard to nucleotide binding, oligomerization and ATPase activity (Beinker *et al.*, 2005 ▶; Werbeck *et al.*, 2009 ▶; Zeymer *et al.*, 2013 ▶). On the basis of this detailed analysis, we are now able to tackle open mechanistic questions concerning nucleotide-state sensing and the conversion of chemical energy from ATP hydrolysis into mechanical motion. We determined high-resolution X-ray structures in different nucleotide states for one of the motor subunits, namely NBD2, of ClpB and combined the structural analysis with nucleotide-binding kinetics studies. Our results provide a better understanding of how the ATPase cycle of the molecular motor is regulated and modulated, which is essential in order to gain further insights into the functioning of the complete disaggregation machine.

## 2. Materials and methods   

### 2.1. Construct design and mutagenesis   

The design of the ClpB NBD2 construct, comprising amino acids 520–854 of ClpB from *T. thermophilus*, has been described previously (Beinker *et al.*, 2005 ▶). The active-site mutations K601Q, R621Q, N709A and R806A were introduced by PCR according to the QuikChange protocol (Agilent Technologies, Santa Clara, California, USA). DNA sequencing was performed by Eurofins MGW Operon (Ebersberg, Germany).

### 2.2. Protein expression and purification   

All ClpB NBD2 variants were expressed recombinantly in *Escherichia coli* BL21 (DE3) RIL cells and purified as described previously (Beinker *et al.*, 2005 ▶; Werbeck *et al.*, 2009 ▶). The proteins were stored in buffer *A* (50 m*M* Tris–HCl pH 7.5, 50 m*M* KCl, 5 m*M* MgCl_2_, 2 m*M* EDTA).

### 2.3. Crystallization and structure determination   

Crystals of all ClpB NBD2 variants were grown in a hanging-drop vapour-diffusion setup at 20°C. The protein solution (∼8 mg ml^−1^ in buffer *A*) was either nucleotide-free or was supplemented with 2 m*M* ADP, 2 m*M* MANT-dADP or 2 m*M* AMPPCP and was mixed in a 1:1 ratio with the reservoir solutions. Crystals of wild-type ClpB NBD2 (space group *P*6_5_) in the nucleotide-free state and in complex with ADP or with AMPPCP grew in 0.2 *M* sodium citrate, 0.1 *M* HEPES–NaOH pH 7.0, 20% 2-propanol. Adding 15%(*v*/*v*) PEG 400 afforded sufficient cryoprotection for flash-cooling in liquid nitrogen. The reservoir solution for the crystallization of ClpB NBD2 K601Q in complex with MANT-dADP (space group *P*2_1_2_1_2_1_) consisted of 0.1 *M* HEPES–NaOH pH 7.5, 12% PEG 6000, 50 m*M* MgCl_2_. The cryoprotectant solution was additionally supplemented with 25% glycerol and 2 m*M* MANT-dADP. Crystals of ClpB NBD2 R621Q in complex with ADP or AMPPCP (space group *P*6_5_) grew in 0.1 *M* Tris–HCl pH 7.5, 15–20% 2-propanol, 10–100 m*M* MgCl_2_. The cryoprotectant solution was additionally supplemented with 15% PEG 400 and 2 m*M* of the respective nucleotide. Crystals were passed quickly through the cryoprotectant solutions and were flash-cooled in liquid nitrogen. Where indicated, crystals of ClpB NBD2 R621Q were soaked in cryoprotectant solution supplemented with 100 m*M* GdmCl for 60 min prior to cooling. Diffraction data collection was performed on beamline X10SA at the Swiss Light Source (Villigen, Switzerland) with the crystals maintained at 100 K. The *XDS* program package was used for data processing (Kabsch, 2010 ▶). Phasing of the ADP-bound wild-type structure was performed by molecular replacement using *CNS* with residues 520–854 of the full-length ClpB structure (PDB entry 1qvr; Lee *et al.*, 2003 ▶) as the search model. Patterson correlation refinement (Brünger, 1990 ▶) was required owing to the different domain arrangement of the search model. Subsequent structures were solved using *Phaser* (McCoy *et al.*, 2007 ▶). Model building and refinement was performed in iterative cycles using *Xfit* (McRee, 1999 ▶) or *Coot* (Emsley & Cowtan, 2004 ▶) and *REFMAC* (Murshudov *et al.*, 2011 ▶) including TLS refinement (Winn *et al.*, 2001 ▶), respectively. The model quality was validated with *MolProbity* (Chen *et al.*, 2010 ▶). Structure illustrations were generated using *PyMOL* (Schrödinger). The coordinates and structure factors have been deposited in the Protein Data Bank (PDB entries 4lj4, 4lj5, 4lj6, 4lj7, 4lj8, 4lj9 and 4lja).

### 2.4. Stopped-flow experiments and global fitting analysis of nucleotide-binding data   

Nucleotide-binding kinetics measurements of ClpB NBD2 variants were performed using a BioLogic SFM-400 stopped-flow instrument (BioLogic Science Instruments, Claix, France) in single mixing configuration at 25°C, essentially as described before for the wild-type protein (Werbeck *et al.*, 2009 ▶). The fluorescently labelled nucleotides MANT-dADP and MANT-dATP were purchased from BIOLOG (Bremen, Germany). The excitation wavelength was 296 nm and the fluorescence signal was observed using a 400 nm longpass filter (400FG03-25, LOT Oriel Group). This setup was used to selectively excite protein-bound MANT-nucleotides *via* fluorescence resonance energy transfer (FRET) from the initially excited tryptophan residues of the protein. Measurements were performed in buffer consisting of 50 m*M* Tris–HCl pH 7.5, 200 m*M* KCl, 5 m*M* MgCl_2_, 2 m*M* EDTA. A set of direct mixing, displacement and competition experiments was performed to determine the association rate constants *k*_on_ and the dissociation rate constants *k*_off_ for both labelled nucleotides (MANT-dADP and MANT-dATP) and unlabelled nucleotides (ADP and ATP). The nucleotide-binding affinity *K*_d_ was calculated from the ratio *k*_off_/*k*_on_. ATP-binding experiments were performed in the presence of 0.01 mg ml^−1^ pyruvate kinase and 200 µ*M* phosphoenol pyruvate as an ATP-regenerating system (MANT-nucleotides are unaffected by pyruvate kinase). Firstly, direct mixing experiments were performed in which 2 µ*M* ClpB NBD2 was mixed with different concentrations (5–25 µ*M*) of MANT-dADP or MANT-dATP. In displacement experiments, 2 µ*M* ClpB NBD2 was incubated with 5 µ*M* MANT-dADP or MANT-dATP and then mixed with different concentrations of ADP (0.5–5 m*M*). In competition experiments, ClpB NBD2 was mixed simultaneously with both MANT-dADP and unlabelled nucleotide (ADP or ATP), with final mixing concentrations of 0.5 µ*M* protein, 2.5 µ*M* MANT-dADP and 2.5–100 µ*M* ADP or 25–300 µ*M* ATP. Additional experimental data were needed to unambiguously determine the ATP-binding parameters. We therefore performed another displacement experiment in which 1 µ*M* ClpB NBD2 was incubated with 200 µ*M* ATP (in the presence of phosphoenol pyruvate and pyruvate kinase as an ATP-regenerating system) and then mixed with 10–100 µ*M* MANT-dADP. The different experimental data sets were combined in a global fitting analysis using the program *DYNAFIT* (Kuzmic, 1996 ▶). The kinetic model given in the scheme below (where Nt = ADP or ATP) was applied for the fitting procedure, which utilizes numerical integration of the corresponding differential equations and nonlinear least-squares minimization.
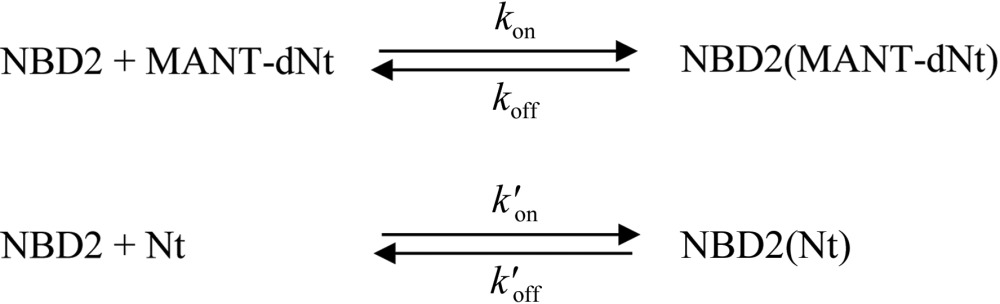


The rate constants were treated as global parameters that were shared by all data sets included; the response coefficients that reflect the amplitude information were treated as semi-global parameters shared among one experimental series.

### 2.5. Steady-state ATPase assay   

The steady-state ATPase activity of ClpB NBD2 variants was measured in a coupled colorimetric assay essentially as described previously (Schlee *et al.*, 2001 ▶; Beinker *et al.*, 2005 ▶). The measurements were performed at 25°C using a Jasco V-­650 spectrophotometer (Jasco Germany GmbH). 10 µ*M* protein was incubated in assay buffer (50 m*M* Tris–HCl pH 7.5, 100 m*M* KCl, 2 m*M* EDTA, 2 m*M* DTE, 0.8 m*M* phosphoenolpyruvate, 0.4 m*M* NADH, 0.1 g l^−1^ BSA, 4 U ml^−1^ pyruvate kinase, 6 U ml^−1^ lactate dehydrogenase, 5 m*M* MgCl_2_). The reaction was started by adding 1 m*M* ATP. The decrease in NADH absorption was monitored at 340 nm over time and the maximum slope was used to calculate the ATPase turnover rate per molecule.

## 3. Results and discussion   

### 3.1. High-resolution structures of ClpB NBD2 in different nucleotide states reveal an inactive, open conformation of the catalytic site   

In order to understand the mechanism behind the force generation upon ATP hydrolysis in the disaggregation machine ClpB, we aimed at obtaining high-resolution crystal structures of one of its two nucleotide-binding domains, NBD2, in different nucleotide states. We sought to determine structural differences between snapshots of different stages during the ATPase cycle in order to reveal the essential conformational changes that drive this molecular motor. We therefore crystallized ClpB NBD2 from *T. thermophilus* under nucleotide-free conditions as well as in the presence of ADP and the nonhydrolysable ATP analogue AMPPCP. Crystallization experiments using 2-propanol as the precipitant yielded well diffracting diamond-shaped crystals for all three states. Structures were determined by molecular replacement to resolutions of 2.8 Å (nucleotide-free), 2.4 Å (ADP) and 1.9 Å (AMPPCP) (Table 1 ▶). The ClpB NBD2 molecules exhibit a helical arrangement with sixfold symmetry owing to the hexagonal screw axis of the crystal lattice. Therefore, the interface between neighbouring NBD2 molecules is slightly shifted compared with a planar hexameric complex, which to date could only be modelled based on electron-microscopy data (Lee *et al.*, 2007 ▶; Biter *et al.*, 2012 ▶) and not observed in X-­ray structures of ClpB. In our structures, the proposed arginine finger Arg747 located in this interface is about 11 Å away from the nucleotide bound to the neighbouring NBD2 molecule. The overall fold of our structures is very similar to recently published structures of ClpB NBD2 (Biter *et al.*, 2012 ▶) and the corresponding part of the structure of full-length ClpB (Lee *et al.*, 2003 ▶). However, we observed a deviation in the positioning of the helix–loop–helix motif comprising residues 710–740, which is most likely to be caused by alternative crystal contacts in our crystal form. Most importantly, our structures of the nucleotide-free and AMPPCP-bound states are of significantly higher resolution than those available previously, allowing a more detailed structural analysis.

**Figure 2:**
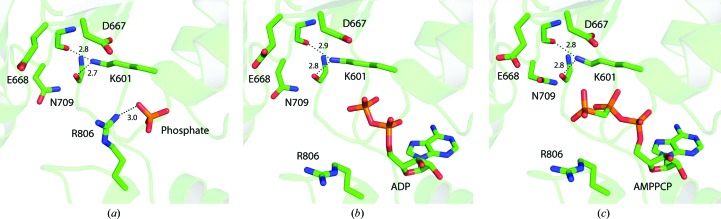
Crystal structures of wild-type ClpB NBD2 in the absence of nucleotide (*a*) and in complex with ADP (*b*) and the nonhydrolysable ATP analogue AMPPCP (*c*). The three structures are highly similar, showing an open and inactive arrangement of the catalytic site, independent of the nucleotide state. Strikingly, the Walker A lysine Lys601 is present in a fully stretched conformation, pointing away from the nucleotide and forming hydrogen bonds with the backbone carbonyl groups of Gly595 and Ser708. This leads to an unfavourable positioning of the catalytically essential residues of the Walker B motif (Asp667 and Glu668) and the sensor 2 motif (Asn709). The sensor 2 residue Arg806 is captured by a crystal contact with the neighbouring NBD2 molecule in both the ADP-bound and AMPPCP-bound forms. In the nucleotide-free state Arg806 interacts with the inorganic phosphate ion bound in the catalytic site. Distances are given in Å.

We focused on analyzing the arrangement of the mechanistically relevant residues in the catalytic site (Fig. 2 ▶). The nucleotide-bound structures contained one molecule of ADP or AMPPCP positioned by the highly conserved hydrogen-bonding network consisting of nucleobase inter­actions as well as hydrogen bonds between the phosphate groups and the P-­loop residues. The nucleotide-free structure contained a phosphate ion bound at the same position as the α-­phosphate in the nucleotide-bound structures. This single phosphate ion forms a salt bridge (*d* = 3.0 Å) with the positively charged side chain of the sensor 2 residue Arg806, which is not observed in the nucleotide-bound states, in which Arg806 is involved in a crystal contact with Glu520 of a neighbouring molecule. Apart from this, the structures of the different nucleotide states are highly similar. Strikingly, in all ClpB NBD2 structures determined to date the side chain of the Walker A lysine Lys601, which is crucial for nucleotide binding and hydrolysis, is present in a completely stretched conformation that prevents the expected interaction with the β/γ-phosphates of the bound nucleotide (Fig. 2 ▶). Instead, the stretched Lys601 side chain forms hydrogen bonds to the carbonyl backbone groups of Gly595 and Ser708. These unusual interactions directly influence both the conformation of the P-loop (Gly595–Thr602) and the essential sensor 1 motif (Asn709). They furthermore stabilize an open, inactive conformation of the catalytic site in which the Walker B motif consisting of Asp667 and Glu668, which is believed to activate the attacking water molecule for ATP hydrolysis, cannot come close enough to fulfil its task and therefore rests in an inactive position. The second remarkable feature observed in all ClpB NBD2 structures is the lack of the essential Mg^2+^ ion usually coordinated by the phosphates of the bound nucleotide. Despite extensive efforts including co-crystallization with up to 100 m*M* MgCl_2_, it was not possible to incorporate the crucial Mg^2+^ ion into the nucleotide-bound structures of wild-type ClpB NBD2. This further supports the hypothesis that the catalytic site arrangements obtained to date reflect an inactive resting state with regard to ATP hydrolysis of ClpB NBD2. Importantly, this inactive picture characterized by a stretched Walker A lysine conformation and the absence of Mg^2+^ from the catalytic site is not only observed in our structures but also in all other ClpB NBD2 structures published to date, independent of the nucleotide state (Biter *et al.*, 2012 ▶; Lee *et al.*, 2003 ▶).

### 3.2. Implications from a ClpB NBD2 Walker A mutant (K601Q) structure in complex with the fluorescently labelled nucleotide MANT-dADP   

**Figure 3:**
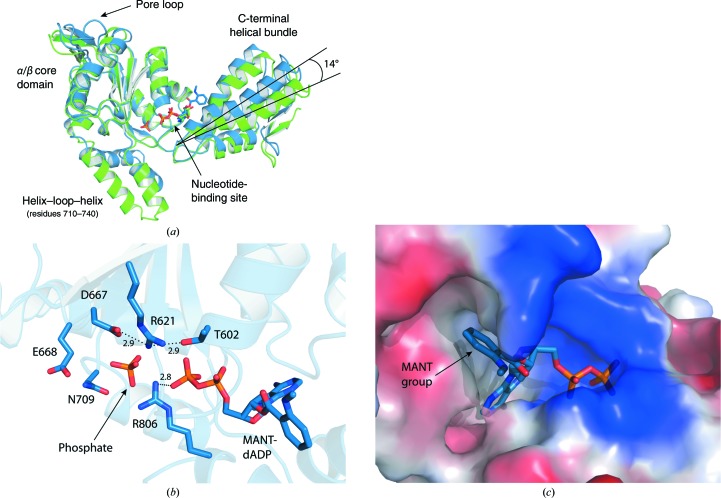
ClpB NBD2 K601Q in complex with MANT-dADP. (*a*) The two different crystal forms obtained for ClpB NBD2. Wild-type protein in complex with ADP, space group *P*6_5_, is shown in green. Walker A mutant K601Q in complex with MANT-dADP, space group *P*2_1_2_1_2_1_, is shown in blue. The cleft between the core domain and the C-terminal helical bundle is narrowed by 14° in the structure of ClpB NBD2 K601Q in complex with MANT-dADP, leading to a slightly more closed catalytic site. There is a large deviation of the helix–loop–helix motif (710–740), which is owing to different crystal contacts. Furthermore, we observe clear electron density for the previously unstructured pore-loop region. (*b*) The catalytic site arrangement of ClpB NBD2 K601Q in complex with MANT-dADP. An inorganic phosphate ion is located 5 Å from the β-phosphate of the bound nucleotide, suggesting an ADP + P_i_ (product-bound) state. The sensor 2 residue Arg806 interacts with the β-phosphate of the nucleotide. The conserved residue Arg621 is located in between the Walker A threonine Thr602 and the Walker B aspartate Asp667 at a position where the essential Mg^2+^ ion is expected to bind. Distances are given in Å. (*c*) The MANT group of the fluorescently labelled nucleotide MANT-dADP fills a mainly hydrophobic cavity in ClpB NBD2, explaining the higher binding affinity of MANT-dADP compared with the unlabelled nucleotide ADP. The electrostatic surface potential is visualized (red, negative charge; blue, positive charge).

In order to perturb the open, inactive arrangement of the ClpB NBD2 catalytic site, we replaced the Walker A lysine with a glutamine. It has previously been shown that this mutation causes severe defects in both nucleotide binding and ATPase activity (Schlee *et al.*, 2001 ▶). To crystallize the mutant ClpB variant NBD2 K601Q, we supplemented the crystallization cocktail with the fluorescently labelled nucleotide MANT-dADP, which has been routinely used for nucleotide-binding studies of ClpB and is known to bind more than 100-fold more strongly to NBD2 than unlabelled ADP (Werbeck *et al.*, 2009 ▶). This compensated for the loss in binding affinity owing to the Walker A mutation and allowed us to obtain a nucleotide-bound structure of ClpB NBD2 K601Q (Fig. 3 ▶).

The complex of ClpB NBD2 K601Q and MANT-dADP crystallized from a condition containing PEG 6000 and yielded a different crystal form to that observed previously for the wild-type protein in conditions containing 2-propanol (*P*2_1_2_1_2_1_ instead of *P*6_5_). The structure was determined by molecular replacement to a resolution of 2.8 Å (Table 1 ▶), showing well defined electron density for the previously unstructured pore-loop region (residues 640–650). The fluorescently labelled MANT-dADP binds at the same position as the unlabelled nucleotides in the structures obtained previously. The aromatic MANT group attached to the 3′-OH of the ribose fits well into a mainly hydrophobic cavity (Fig. 3 ▶ *c*), which also explains the preferential binding of 3′-MANT-ADP compared with 2′-MANT-ADP owing to steric restrictions (Werbeck *et al.*, 2009 ▶). The catalytic site shows a more closed conformation, characterized by a narrowing of the cleft between the large α/β core domain and the C-terminal small domain (Fig. 3 ▶ *a*). A domain-motion analysis using *DynDom* (Hayward & Lee, 2002 ▶) comparing the MANT-dADP-bound form with the ADP-bound form showed that the angle between the domains decreases by 14°. The essential sensor 2 residue Arg806 located on the C-­terminal small domain forms a salt bridge (*d* = 2.8 Å) with the β-phosphate of the nucleotide. An additional phosphate ion is present in the active site at a distance of 5 Å from the β-­phosphate of the nucleotide, suggesting that we may have captured an ADP + P_i_ (product-bound) state here. However, this structure also does not reveal the active arrangement of the essential Walker B motif that would explain its mode of action. Furthermore, we were again not able to observe the crucial Mg^2+^ ion in the catalytic site. Interestingly, another highly conserved active-site residue, Arg621, adopts a different conformation to that in the structures described previously. This arginine points towards the nucleotide and is positioned directly between the Walker A residue Thr602 and the Walker B residue Asp667, thereby causing an arrangement that might block the incorporation of Mg^2+^ because it places a positive charge close to where the positively charged Mg^2+^ ion is expected to bind (Fig. 3 ▶ *b*). With the intention to facilitate the incorporation of Mg^2+^ and thus obtain an active structure, we next generated the mutant variant ClpB NBD2 R621Q and performed both biochemical characterization and crystallization experiments, which are described in the following sections.

### 3.3. The active-site mutations R806A and R621Q modify the discrimination between ADP and ATP binding and influence the ATPase activity of ClpB NBD2   

Changes in nucleotide-binding properties owing to active-site mutations provide very useful insights regarding the mechanistic roles of the respective residues in ATP hydrolysis and nucleotide-state sensing. The nucleotide-binding parameters of ClpB NBD2 have been characterized previously using transient kinetics experiments with fluorescently labelled MANT-nucleotides (Werbeck *et al.*, 2009 ▶). A set of stopped-flow experiments including direct mixing, displacement and competition experiments in combination with global fitting analysis allowed the determination of the binding parameters for both MANT-labelled as well as unlabelled nucleotides. Interestingly, the binding affinity of ClpB NBD2 for ADP is almost 200-fold higher than for ATP. This strong discrimination is mainly owing to a lower dissociation rate constant *k*_off_ for ADP (Werbeck *et al.*, 2009 ▶). However, the X-­ray structures available to date could not explain this phenomenon.

**Figure 4:**
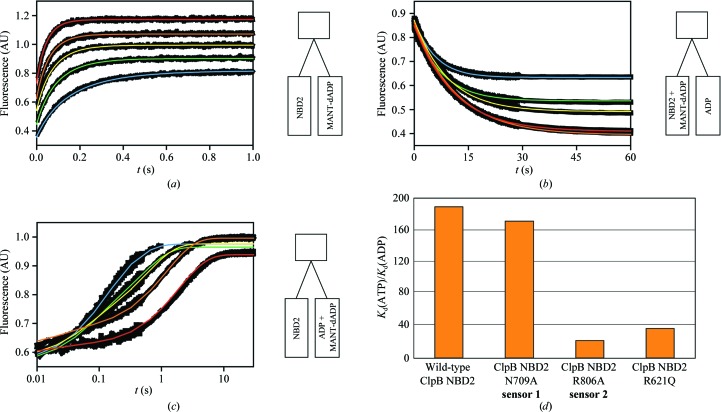
Nucleotide-binding kinetics measurements. The nucleotide-binding stopped-flow experiments were performed as described in §[Sec sec2.4]2.4 with subsequent global data-fitting analysis according to the model given in (1). The nucleotide-binding properties of wild-type ClpB NBD2 and the N709A, R806A and R621Q mutants for MANT-dADP, MANT-dATP, ADP and ATP were determined. The kinetic traces shown exemplarily in this figure correspond to direct mixing (*a*), displacement (*b*) and competition (*c*) experiments using ClpB NBD2 R806A, MANT-dADP and ADP. Coloured lines represent the fits from the global data-fitting procedure. Concentrations increase through blue, green, yellow, orange to red. The schematic experimental setup with the solutions being mixed in the different experiments is depicted next to the kinetic traces. (*a*) Direct mixing of 2 µ*M* NBD2 and 5–25 µ*M* MANT-dADP. (*b*) Displacement experiment: 2 µ*M* ClpB NBD2 was incubated with 5 µ*M* MANT-dADP and then mixed with 0.5–5 m*M* ADP. (*c*) ClpB NBD2 was mixed simultaneously with both MANT-dADP and ADP, giving final mixing concentrations of 0.5 µ*M* protein, 2.5 µ*M* MANT-dADP and 2.5–100 µ*M* ADP. The time axis is plotted on a logarithmic scale to account for the large range of rates in this experimental series. (*d*) The discrimination between ADP and ATP binding is illustrated by the ratio *K*_d_(ATP)/*K*_d_(ADP). The active-site mutations R806A and R621Q significantly reduce the discrimination, primarily by impairing ADP binding. All nucleotide-binding parameters obtained from these experiments are listed in Table 2 ▶.

The residues Asn709 and Arg806 are part of the conserved sensor 1 and sensor 2 motifs, respectively, which have been proposed to be involved in nucleotide-state sensing (Wendler *et al.*, 2012 ▶). We therefore characterized the nucleotide-binding properties and the steady-state ATPase activity of the two sensor-motif mutants NBD2 N709A and NBD2 R806A as well as the mutant NBD2 R621Q that was used for crystallization (Fig. 4 ▶ and Table 2 ▶). Mutating the sensor 1 residue Asn709 caused only very minor changes in nucleotide binding, whereas the ATPase activity was reduced by about 30-fold compared with the wild type. This indicates that Asn709 is of catalytic importance, but is not necessarily directly involved in nucleotide binding, which is in agreement with a previous study on the sensor 1 motif in Hsp104, the yeast homologue of ClpB (Hattendorf & Lindquist, 2002*a* ▶).

Despite being nucleotide-binding competent, the ATPase activity of the sensor 2 mutant NBD2 R806A was almost completely abolished. This is in agreement with previous studies on other AAA+ proteins, which showed that the sensor 2 arginines are catalytically essential, most likely by stabilizing intermediate states of the hydrolysis cycle (Wendler *et al.*, 2012 ▶). Our nucleotide-binding kinetics measurements revealed a significantly reduced discrimination between ADP and ATP binding for the sensor 2 mutant NBD2 R806A, with only a very minor influence on ATP binding arising from the mutation, but significantly impaired ADP binding (Fig. 4 ▶ *d*). This result hints towards a direct interaction of Arg806 with the nucleotide that contributes to the binding energy in the ADP state but not in the ATP state, which is discussed in further detail in the following section with respect to the structural data available. Previous results from equilibrium titrations with Hsp104 by Lindquist and coworkers suggested similarly impaired nucleotide binding for both ADP and ATP in the presence of the sensor 2 mutation R826M in the oligomeric context of the full-length protein (Hattendorf & Lindquist, 2002*b* ▶). Our approach using only the isolated nucleotide-binding domain 2 (NBD2) of ClpB allows a more detailed kinetic analysis of the nucleotide-binding behaviour that is able to capture the differences in nucleotide discrimination.

The mutant variant NBD2 R621Q is nucleotide-binding competent, but shows an approximately 30-fold reduced ATPase activity. It is highly likely that Arg621 is involved in stabilizing negative charges, especially in the transition state. The R621Q mutation significantly reduces the discrimination between ATP and ADP, mainly by impaired ADP binding, but also owing to a slight increase in ATP binding (Fig. 4 ▶ *d* and Table 2 ▶). As discussed before for NBD2 R806A, it is possible that Arg621 functions as a nucleotide sensor, adopting alternative conformations dependent on the nucleotide-binding state. Arg621 is highly conserved in ClpB and Hsp104 homologues from different organisms, as well as related AAA+ proteins that possess a second C-terminal nucleotide-binding domain, such as ClpA and ClpC. A potential mechanistic role of Arg621 is discussed further in the next section, which introduces the high-resolution X-ray structure of ClpB NBD2 R621Q in complex with the nonhydrolysable ATP analogue AMPPCP.

### 3.4. Mechanistic insights from the structure of ClpB NBD2 R621Q bound to an ATP analogue with the essential Mg^2+^ ion incorporated into the catalytic site   

#### 3.4.1. The role of Arg621 in Mg^2+^ incorporation   

**Figure 5:**
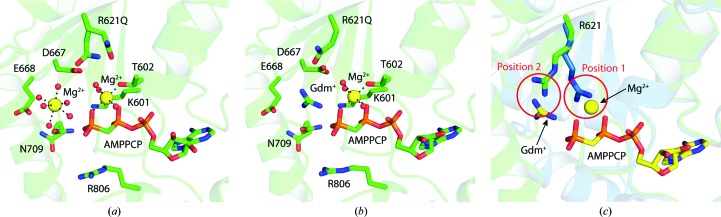
The active-site mutation R621Q facilitates the incorporation of Mg^2+^ into ClpB NBD2. (*a*) Structure of ClpB NBD2 R621Q in complex with AMPPCP. The essential Mg^2+^ ion is coordinated by the β- and γ-phosphates of the bound nucleotide, the Walker A threonine Thr602 and three water molecules. Owing to the mutation and the high MgCl_2_ concentration of 75 m*M* in the crystallization solution, a second Mg^2+^ ion with a complete hydration shell [*d*(Mg–O) = 2.0–2.3 Å] is present in the catalytic site at a distance of 4.5 Å from the γ-phosphate. The Walker A lysine Lys601 adopts the stretched conformation observed in the previous structures and does not contact the nucleotide. The Walker B aspartate Asp667 forms hydrogen bonds to water molecules of the hydration shell of the essential Mg^2+^ ion. However, there is no significant rearrangement of the catalytic site compared with the Mg-free structures. Residues 621 and 709 were modelled using alternative conformations, which are both shown in the figure. (*b*) Structure of ClpB NBD2 R621Q in complex with AMPPCP and GdmCl. Using a lower MgCl_2_ concentration of 10 m*M* and additionally soaking with 100 m*M* GdmCl, the second Mg^2+^ ion was replaced by a Gdm^+^ ion, whereas the essential Mg^2+^ ion bound at the phosphates of the nucleotide remained. The Gdm^+^ ion represents a mobile arginine side-chain mimic, suggesting that the mutated Arg621 would usually occupy this position. Residue 709 was modelled using alternative conformations, which are both shown in the figure. (*c*) Superposition of the two crystal forms observed for ClpB NBD2 showing two different positions for the conserved active-site residue Arg621 (green, wild-type NBD2 + AMPPCP; blue, NBD2 K601Q + MANT-dADP). Additionally, the AMPPCP, the essential Mg^2+^ ion and the Gdm^+^ ion of the NBD2 R621Q structure shown in (*b*) are overlaid in yellow, illustrating the two possible positions of the Arg621 side chain. In position 1, Arg621 is located close to the nucleotide and impairs Mg^2+^ incorporation. In position 2, it is at a greater distance from the nucleotide, allowing Mg^2+^ binding.

Analysis of the ClpB NBD2 K601Q structure (see Fig. 3 ▶ *b*) led to the hypothesis that the highly conserved residue Arg621 might interfere with the binding of Mg^2+^ to the phosphates of the bound nucleotide in the catalytic site. In at least one of its possible conformations, the Arg621 side chain positions a positive charge close to the expected Mg^2+^-binding site and thereby competes with Mg^2+^ incorporation. The mutation R621Q was therefore introduced to facilitate the incorporation of the essential Mg^2+^ into the ClpB NBD2 structure. Crystallization of ClpB NBD2 R621Q in the presence of the nonhydrolysable ATP analogue AMPPCP and MgCl_2_ yielded well diffracting diamond-shaped crystals in space group *P*6_5_ (Table 1 ▶). The structure was determined to a resolution of 1.7 Å and for the first time we could unambiguously locate the essential Mg^2+^ ion between the β- and γ-phosphates of the bound AMPPCP (Fig. 5 ▶ *a*). The Mg^2+^ ion is coordinated octahedrally by the two phosphate groups, the hydroxyl group of the Walker A residue Thr602 and three water molecules. The Walker B residue Asp667 forms hydrogen bonds to two of the hydration-shell water molecules. Additionally, we observe electron density for a second Mg^2+^ ion coordinated octahedrally by six water molecules at a distance of 4.5 Å to the γ-­phosphate of the bound AMPPCP. We assume that this additional Mg^2+^ ion is not of mechanistic relevance but rather is incorporated owing to the R621Q mutation and the non­physiologically high MgCl_2_ concentration of 75 m*M* used in the crystallization cocktail. Indeed, when comparing the Arg621 side-chain conformations in the different structures available, two alternative positions are observed. The positively charged side-chain guanidinium group is either located close to the nucleotide, where it blocks the incorporation of the essential Mg^2+^ ion, or close to where the second (most likely nonphysiological) Mg^2+^ ion is found in the NBD2 R621Q structure (Fig. 5 ▶ *c*). The negatively charged environment that surrounds this second position (formed by Asp623, Asp667 and Glu668) can easily stabilize a positive charge, be it the Arg621 side chain in the wild-type protein or the second Mg^2+^ ion incorporated into this position owing to the R621Q mutation. To verify this hypothesis, we repeated the crystallization experiment in the presence of only 10 m*M* MgCl_2_ and additionally soaked with 100 m*M* guanidinium chloride (GdmCl), which was supposed to function as a mobile substitute for the arginine side chain that was removed owing to the R621Q mutation. Indeed, in the structure obtained from crystals grown in this condition, only one Mg^2+^ ion is present, which occupies the expected position between the β- and γ-­phosphates of the bound AMPPCP. Additionally, a Gdm^+^ ion is localized where the second Mg^2+^ ion was bound before, suggesting that this position would usually be occupied by the Arg621 side chain (Fig. 5 ▶ *b*).

Based on this structural analysis and the kinetic data showing a reduced discrimination between ADP and ATP binding in ClpB NBD2 R621Q (Fig. 4 ▶ *d* and Table 2 ▶), we propose that Arg621 is involved in nucleotide sensing and the incorporation of the essential Mg^2+^ ion into the catalytic site which is crucial for efficient ATP hydrolysis. Arg621 adopts two different conformations, which are presumably dependent on the stage of the ATPase cycle (Fig. 5 ▶ *c*). Since the R621Q mutation selectively impairs ADP binding, we conclude that in the ADP state Arg621 contributes significantly to the nucleotide-binding energy, presumably by mimicking the nucleotide-associated Mg^2+^. In the ATP state, Arg621 is most likely to adopt the position further away from the nucleotide, thereby not contributing to the binding energy but allowing the incorporation of the essential Mg^2+^ ion. Regulatory metal switches have also been found in other ATPases, such as the DNA mismatch-repair protein MutS, in which the essential Walker B motif is rearranged in the absence of Mg^2+^ (Lebbink *et al.*, 2010 ▶), which is reminiscent of the situation that we observe for ClpB NBD2. We therefore hypothesize that a variation of a metal-switch mechanism with Arg621 as a key regulatory feature is implemented in ClpB NBD2.

#### 3.4.2. Nucleotide state-sensing mechanisms   

**Figure 6:**
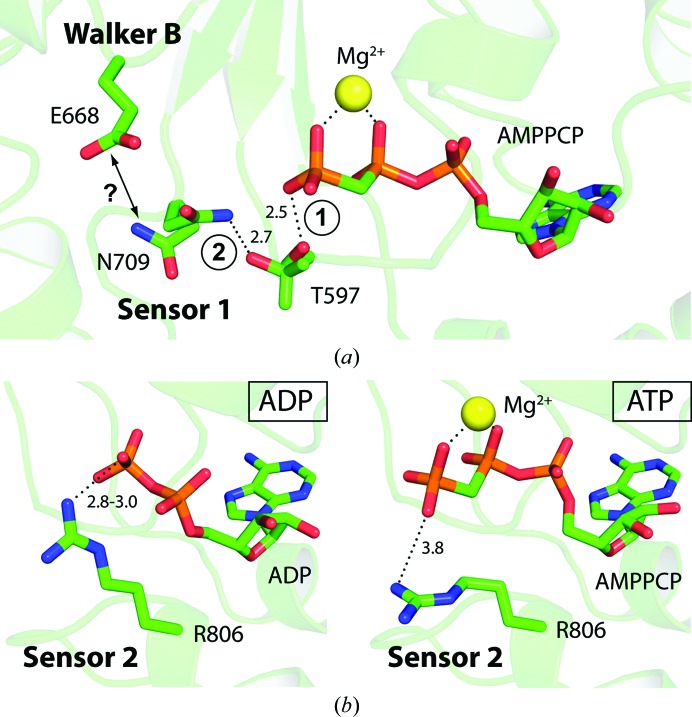
Nucleotide-state sensing in ClpB NBD2. (*a*) Sensor 1 motif: structure of ClpB NBD2 R621Q in complex with AMPPCP showing alternative conformations for the P-loop residue Thr597 and the sensor 1 residue Asn709, which are involved in a hydrogen-bonding network around the nucleotide. Depending on the nucleotide state, Thr597 can form a hydrogen bond to either the γ-phosphate of ATP (1) or, in the absence of the γ-phosphate, to sensor 1 residue Asn709 (2). Currently, it is unclear how such a switch is further communicated through the molecule. However, it has been proposed previously that the sensor 1 motif is involved in hydrolysis, coordinating the attacking water together with the Walker B glutamate Glu668 (Gai *et al.*, 2004 ▶). This might only be possible when Asn709 is not captured in the interaction with Thr597; thus, in the ATP-bound state. Distances are given in Å. (*b*) Sensor 2 motif: ClpB NBD2 in the ADP and ATP state, respectively, showing different positions of the sensor 2 residue Arg806, which is not influenced by crystal contacts in the structures shown in this figure. In the ADP state (left panel; ClpB NBD2 R621Q + ADP), Arg806 interacts with the β-­phosphate of the nucleotide (*d* = 3.0 Å), which is also the case in the MANT-dADP-bound structure (*d* = 2.8 Å) shown in Fig. 3 ▶(*b*) and in another recently published ADP-bound NBD2 structure (*d* = 2.8 Å; Biter *et al.*, 2012 ▶). This direct interaction between Arg806 and the nucleotide in the ADP state contributes to the nucleotide-binding energy, which is in agreement with the observation that the R806A mutation causes impaired ADP binding (Table 2 ▶). In the ATP state (right panel; ClpB NBD2 R621Q + AMPPCP + GdmCl), Arg806 must change its position because it would otherwise clash with the γ-phosphate. Indeed, it bends away from the phosphates (*d* = 3.8 Å), thereby no longer contributing to the nucleotide binding energy significantly, which is supported by our nucleotide-binding kinetics measurements showing that the R806A mutation has no significant effect on ATP binding (Table 2 ▶). The clearly different behaviour of Arg806 in the presence of ADP compared with ATP demonstrates that this residue functions as a *bona fide* nucleotide sensor.

The sensor 1 and 2 motif residues Asn709 and Arg806, respectively, are catalytically essential and have been proposed to be involved in sensing and communicating different nucleotide states (Guenther *et al.*, 1997 ▶). The structural basis of how this function is fulfilled, however, is poorly understood. Owing to the high resolution of 1.7 Å, we were able to assign alternative conformations for certain residues in the structure of ClpB NBD2 R621Q in complex with AMPPCP. We identified a hydrogen-bonding network between sensor 1 residue Asn709, P-loop residue Thr597 and the γ-phosphate of the bound nucleotide. Depending on its orientation, Thr597 can either form a hydrogen bond to the nucleotide or to the side chain of Asn709 (Fig. 6 ▶ *a*). One can speculate that in the ATP state Thr597 preferentially interacts with the γ-phosphate. In the nucleotide-free or ADP state, when no γ-phosphate is present, it rotates and interacts with Asn709. This putative switch might be of regulatory importance. Even though the sensor 1 mutation N709A causes no significant changes in the nucleotide-binding properties, it severely impairs ATP hydrolysis. This is in agreement with the hypothesis that the sensor 1 residue works in concert with the Walker B motif in activating the attacking water molecule during hydrolysis (Gai *et al.*, 2004 ▶). A nucleotide-dependent conformational switch of Asn709 might be the regulating element for this function. However, the structural data available are not sufficient to unambiguously confirm this hypothesis. Based on the kinetic data, we can state that the essential sensor 1 motif (Asn709) is not directly involved in nucleotide binding, which is inconsistent with the role of a classic nucleotide sensor. This result is in agreement with a previous study by Hattendorf & Lindquist (2002*a* ▶).

In contrast, our characterization of the sensor 2 motif (Arg806) provides clear evidence for direct nucleotide sensing and discrimination. In addition to the AMPPCP-bound structure of ClpB NBD2 R621Q in the presence of MgCl_2_ and GdmCl, we also obtained the structure of this mutant variant in the ADP-bound state to a resolution of 2.1 Å (Table 1 ▶). Since the sensor 2 residue Arg806 was no longer trapped in a crystal contact in these structures, we could identify two different conformations of Arg806 that are adopted depending on the nucleotide state (Fig. 6 ▶ *b*). In the ADP state, the side chain of Arg806 interacts directly with the β-phosphate of the bound nucleotide (*d* < 3.0 Å), which is also observed in both our MANT-dADP-bound structure and the recently published NBD2 structure (PDB entry 4fcw; Biter *et al.*, 2012 ▶). In the ATP state, the Arg806 side chain must adopt an alternative conformation because otherwise it would clash with the γ-­phosphate. Indeed, Arg806 is located about 4 Å away from the nucleotide in the AMPPCP-bound structure. This is in agreement with our nucleotide-binding data, which show that the sensor 2 mutation R806A causes significantly impaired ADP binding but almost no change in ATP binding. It can be concluded that the direct interaction with the nucleotide in the ADP state contributes to the binding energy, whereas in the ATP state no interaction between Arg806 and the nucleotide takes place. This, together with the function of residue Arg621 described in the previous sections, provides a veritable explanation for the observed discrimination between ADP and ATP binding in ClpB NBD2. The combination of structural and kinetic analysis further defines the role of the sensor 2 residue Arg806 in nucleotide-state sensing and identifies one clear difference between the ADP and ATP state, which is crucial for understanding the mode of action of this ATPase motor domain.

### 3.5. Comparison with other AAA+ protein structures regarding the hexameric context   

To evaluate the conclusions drawn from the ClpB NBD2 structures presented in this work, it is crucial to discuss the results in the light of the oligomeric context of the holo­protein. Even though smaller oligomeric species, as present for the separate NBD2 construct, hydrolyze ATP efficiently, full disaggregation activity is most likely to require a hexameric assembly. There are several structural studies available that show hexameric states of AAA+ proteins, for example for p97, ClpX, SV40 LTag and ClpC. Despite a common overall architecture, the essential interactions and resulting structural changes in the oligomeric arrangement upon ATP hydrolysis vary remarkably for the different AAA+ proteins. For ClpC, which shares high sequence identity with ClpB, a planar hexameric state in complex with the adaptor protein MecA has been observed in both X-ray structures as well as cryo-EM studies (Liu *et al.*, 2013 ▶; Wang *et al.*, 2011 ▶). Unfortunately, the resolution is not sufficient to discuss detailed side-chain interactions. For the AAA+ helicase LTag from SV40, structures of the planar hexamer are available for different nucleotide states at very high resolution (up to 1.9 Å), which allowed a detailed mechanistic analysis (Gai *et al.*, 2004 ▶). Here, a conserved lysine from the neighbouring subunit works in concert with the classic arginine finger. Furthermore, extensive mechanistic and structural studies regarding the hexameric assembly have been performed for ClpX, another prominent AAA+ unfoldase (Glynn *et al.*, 2009 ▶, 2012 ▶; Martin *et al.*, 2005 ▶; Stinson *et al.*, 2013 ▶), and for p97, an ERAD-associated AAA+ protein (Davies *et al.*, 2008 ▶; Li *et al.*, 2012 ▶; Nishikori *et al.*, 2011 ▶).

To evaluate our results, we compared the average distances between the nucleotide and the catalytically relevant Walker and sensor residues in our structures with those observed in the available high-resolution hexameric AAA+ structures, using the analysis presented by Wendler and coworkers as a starting point (Wendler *et al.*, 2012 ▶). For instance, the distance between the side-chain carbonyl O atom of the Walker B aspartate and the γ-phosphate O atom of the nucleotide ranges from 3.0 to 5.5 Å in published hexameric structures and is 4.5 Å in the case of ClpB NBD2 R621Q + AMPPCP. Similarly, the distance between the nucleotide and the sensor 1 asparagine side chain ranges from 3.1 Å for SV40 LTag (PDB entry 1svm; Gai *et al.*, 2004 ▶) to 6.0 Å for p97 (PBD entry 3cf1; Davies *et al.*, 2008 ▶), and is 4.6 Å in the case of ClpB NBD2. Sensor 2 motifs are only present in a subset of AAA+ proteins. We compared ClpB NBD2 with ClpX (PDB entry 3hws; Glynn *et al.*, 2009 ▶) and also found similar distances and orientations regarding the sensor 2 arginine. Even though we cannot exclude that our single NBD2 structures somewhat differ from their counterparts in the hexamer, this comparison indicates that they may very well represent snapshots of relevant states that are also adopted in the hexameric context.

It is remarkable that all of the structures of ClpB (full-length and NBD2) available to date show a helical arrangement of subunits representing a spiral-like oligomeric state, which is also observed in the available high-resolution structure of ClpA (Guo *et al.*, 2002 ▶), a closely related unfoldase that also bears two NBDs per molecule. Even though cryo-EM data suggest that the planar hexameric ring is the predominant active form, it cannot be excluded that spirals are present in solution and are of certain mechanistic relevance. A possible biological role of a spiral has actually been discussed for other AAA+ proteins (Erzberger & Berger, 2006 ▶).

**Figure 7:**
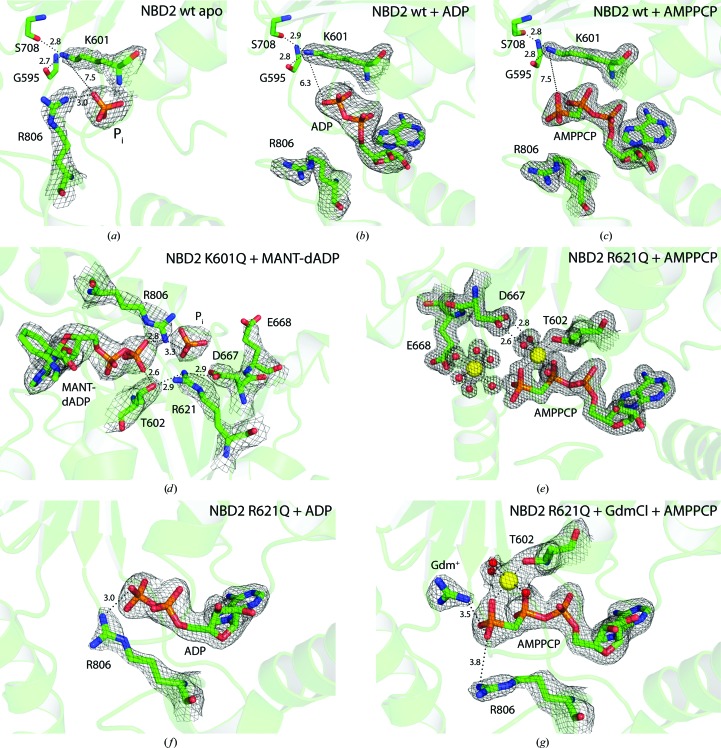
Overview of the states captured for ClpB NBD2. 2*mF*_o_ − *DF*_c_ electron density (contour levels are given below) is shown for relevant parts of each structure, such as the bound nucleotide and conserved Walker or sensor residues. Mg^2+^ ions are shown in yellow, including their octahedral coordination sphere with *d*(Mg–O) = 2.0–2.3 Å. Water molecules are shown as red spheres. Distances are given in Å. (*a*) Wild-type ClpB NBD2, nucleotide-free, PDB entry 4lj4, contour level 1.1σ. (*b*) Wild-type ClpB NBD2 in complex with ADP, PDB entry 4lj5, contour level 1.5σ. (*c*) Wild-type ClpB NBD2 in complex with AMPPCP, PDB entry 4lj6, contour level 1.5σ. (*d*) ClpB NBD2 K601Q in complex with MANT-dADP, PDB entry 4lj7, contour level 1.1σ. (*e*) ClpB NBD2 R621Q in complex with AMPPCP, PDB entry 4lj9, contour level 1.5σ. (*f*) ClpB NBD2 R621Q in complex with ADP, PDB entry 4lj8, contour level 1.5σ. (*g*) ClpB NBD2 R621Q in complex with AMPPCP and GdmCl, PDB entry 4lja, contour level 1.5σ. For detailed structure analyses, compare Figs. 2 ▶, 3 ▶, 5 ▶ and 6 ▶.

Even though the exact arrangement of the functionally relevant oligomeric state in ClpB remains elusive, our NBD2 structures contribute to the overall mechanistic understanding by providing structural details at high resolution for one separate motor building block of an AAA+ machine in different states. A summary of the different states captured for ClpB NBD2 is presented in Fig. 7 ▶.

### 3.6. Various P-loop proteins exhibit similar inactive conformations: a potential regulatory function of the Walker A lysine as a molecular switch   

**Figure 8:**
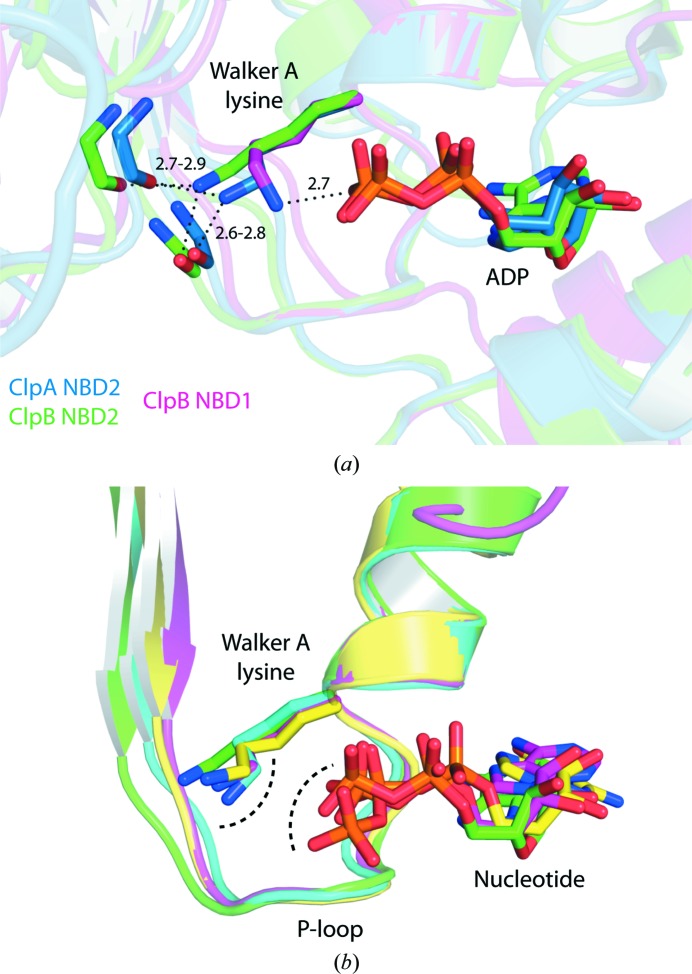
Various P-loop protein structures exhibit an inactive catalytic site conformation. (*a*) P-loop superposition of ClpA NBD2 (blue, PDB entry 1r6b; Xia *et al.*, 2004 ▶) and ClpB NBD2 (green) in complex with ADP. The closely related AAA+ protein ClpA shows an almost identical catalytic site conformation as ClpB, characterized by a stretched Walker A lysine in NBD2 that contacts carbonyl backbone groups. Notably, the corresponding Walker A lysine in NBD1 of both proteins directly interacts with the nucleotide in the conventional manner, which is shown for ClpB NBD1 (pink, PDB entry 4hse; Zeymer *et al.*, 2013 ▶). (*b*) P-loop superposition of nucleotide-bound structures of different P-loop proteins: ClpB NBD2 (green), the kinesin family member Kin10 (pink; PDB entry 3pxn; Cochran *et al.*, 2011 ▶), the human GTPase GIMAP2 that controls apoptosis in lymphocytes (yellow; PDB entry 2xtn; Schwefel *et al.*, 2010 ▶) and the DNA mismatch-repair protein MutS (blue; PDB entry 3k0s; Lebbink *et al.*, 2010 ▶). The Walker A lysine residues adopt a stretched conformation and interact with carbonyl backbone groups but not with the nucleotide, thereby stabilizing an open inactive conformation of the catalytic site.

Even though we were finally able to incorporate the essential Mg^2+^ ion into the structure of ClpB NBD2, the overall conformation of the catalytic site remained open and therefore inactive. The Walker A lysine Lys601 is still stretched and contacts P-loop backbone carbonyl groups but not the nucleotide. We therefore analyzed the structures of other P-­loop proteins available at sufficiently high resolutions and found more examples of such open inactive catalytic site conformations characterized by a stretched Walker A lysine. In the structure of a closely related AAA+ protein, ClpA, the situation is almost identical (PDB entry 1r6b; Xia *et al.*, 2004 ▶). Whereas in NBD1 of both of the proteins ClpA and ClpB the Walker A lysine directly interacts with the nucleotide in the conventional way, in NBD2 the stretched Walker A lysine conformation is observed (Fig. 8 ▶ *a*). However, more distant relatives in the large family of P-loop proteins were also found to adopt this open inactive conformation of the catalytic site in their respective crystal structures. Three examples are shown in Fig. 8 ▶(*b*): the kinesin family member Kin10 in complex with ADP (PDB entry 3pxn; Cochran *et al.*, 2011 ▶), a human GTPase controlling apoptosis in lymphocytes, GIMAP2, in complex with GTP (PDB entry 2xtn; Schwefel *et al.*, 2010 ▶) and the DNA mismatch-repair protein MutS in complex with ADP (PDB entry 3k0s; Lebbink *et al.*, 2010 ▶). Interestingly, in the latter example the inactive catalytic site arrangement is also coupled to the absence of Mg^2+^. We hypothesize that the open catalytic site conformations represent an inactive resting state during the ATPase cycle, which is often captured in X-ray structures and may be of regulatory importance. Zhang and Wigley proposed previously that a so-called ‘glutamate switch’ directly controls the conformation of the essential Walker B glutamate residue in AAA+ domains, keeping it in an inactive state by hydrogen bonding to a polar residue located on the tip of β-strand β2 (Zhang & Wigley, 2008 ▶). In ClpB NBD2 from *T. thermophilus*, this residue corresponds to a threonine (Thr625). However, we do not observe a direct hydrogen-bonding interaction with the Walker B glutamate Glu668 in our structures. Instead, we suggest a different mechanism that forces the catalytically essential residues, including the Walker B glutamate, into an inactive position. We propose that the Walker A lysine in its stretched conformation acts like a ‘molecular doorstop’ that keeps the catalytic site open and thereby inactive in order to avoid unnecessary ATP turnover, for example in the absence of a secondary substrate. Many ATPases have a low basal ATP-hydrolysis activity which is significantly stimulated upon addition of substrate, with the highly conserved Walker A lysine having a catalytic role. We propose a second, regulatory function for the Walker A lysine, where it is involved in switching between an inactivated, ‘fuel-saving’ state and the conventional active state of a molecular motor.

The question arises as to why this regulatory mechanism is only implemented in a subset of AAA+ domains. In ClpB, the stretched Walker A lysine conformation is observed exclusively in the C-terminal nucleotide-binding domain NBD2, but not in the N-terminal NBD1. In the current mechanistic view, NBD2 is the actual motor of the ClpB disaggregation machine, whereas NBD1 is mainly of regulatory importance, interacting with the co-chaperone DnaK *via* the unique coiled-coil middle domain, which turns out to be a highly sophisticated regulatory element of ClpB (Rosenzweig *et al.*, 2013 ▶; Oguchi *et al.*, 2012 ▶). Thus, NBD1 is already highly regulated, whereas the strong motor NBD2 needs efficient regulation, which we propose is provided by a conformational switch of the Walker A lysine that is likely to be induced by binding of the substrate protein.

## 4. Conclusions   

Our structure-based analysis of ClpB NBD2, a representative of the AAA+ motor domains, reveals mechanistic insights into nucleotide sensing and the regulation of the ATPase activity. We show how the conserved sensor 2 motif directly contributes to the discrimination between ADP and ATP binding. Furthermore, we suggest that a conserved active-site arginine regulates the incorporation of the essential Mg^2+^ ion and is therefore essential for the functioning of the ATPase cycle. In addition, we propose a regulatory switch where the conserved Walker A lysine, when present in a fully stretched conformation, causes an inactive arrangement of the catalytic site to avoid unnecessary ATPase turnover.

## Supplementary Material

PDB reference: ClpB NBD2, wild type, nucleotide-free, 4lj4

PDB reference: wild type, complex with ADP, 4lj5

PDB reference: wild type, complex with AMPPCP, 4lj6

PDB reference: K601Q, complex with MANT-dADP, 4lj7

PDB reference: R621Q, complex with ADP, 4lj8

PDB reference: R621Q, complex with AMPPCP, 4lj9

PDB reference: R621Q, complex with AMPPCP and guanidinium chloride, 4lja

## Figures and Tables

**Table 1:** Crystallographic data-collection and refinement statistics Values in parentheses are for the highest resolution shell. n.a., not applicable.

	Wild-type ClpB NBD2			ClpB NBD2 K601Q	ClpB NBD2 R621Q		
	Nucleotide-free	ADP	AMPPCP	MANT-dADP	ADP	AMPPCP	AMPPCP + GdmCl
Data collection							
Wavelength (Å)	0.9495	1.01096	0.979259	0.9787	0.9170	1.0000	0.9120
Space group	*P*6_5_	*P*6_5_	*P*6_5_	*P*2_1_2_1_2_1_	*P*6_5_	*P*6_5_	*P*6_5_
Unit-cell parameters							
*a* (Å)	74.5	76.1	74.1	65.7	74.3	74.6	74.8
*b* (Å)	74.5	76.1	74.1	103.9	74.3	74.6	74.8
*c* (Å)	118.1	120.5	118.7	159.1	119.5	119.7	119.5
α (°)	90	90	90	90	90	90	90
β (°)	90	90	90	90	90	90	90
γ (°)	120	120	120	90	120	120	120
No. of molecules per asymmetric unit	1	1	1	3	1	1	1
Resolution (Å)	20–2.8 (3.0–2.8)	20–2.4 (2.5–2.4)	25–1.9 (2.0–1.9)	50–2.8 (2.9–2.8)	44–2.1 (2.2–2.1)	44–1.7 (1.8–1.7)	44–2.0 (2.1–2.0)
No. of unique reflections	9176 (1708)	17302 (2064)	28958 (4115)	48302 (4066)	21813 (2834)	41388 (6484)	25581 (3479)
*R*_merge_ † (%)	0.074 (0.534)	0.085 (0.774)	0.067 (0.473)	0.084 (0.557)	0.053 (0.395)	0.053 (0.387)	0.043 (0.821)
〈*I*/σ(*I*)〉	22.1 (4.3)	10.1 (2.1)	14.1 (3.8)	11.2 (2.2)	19.4 (3.2)	14.7 (3.2)	29.1 (3.0)
Completeness (%)	99.9 (100.0)	98.4 (98.6)	99.9 (100.0)	93.1 (78.3)	99.9 (99.9)	99.9 (100.0)	100.0 (100.0)
Average multiplicity	7.6 (7.7)	5.5 (5.0)	4.8 (3.8)	3.5 (3.5)	6.4 (4.2)	4.8 (5.0)	10.3 (10.6)
Wilson *B* factor (Å^2^)	68.7	54.8	25.6	81.6	40.4	26.7	41.1
Refinement							
*R*_work_ ‡/*R*_free_ §	0.254/0.308	0.235/0.272	0.217/0.262	0.240/0.295	0.232/0.286	0.208/0.244	0.217/0.263
No. of atoms							
Protein	2531	2534	2556	7167	2555	2706	2555
Nucleotide	n.a.	27	31	108	27	31	31
Mg^2+^	n.a.	n.a.	n.a.	n.a.	n.a.	2	1
Gdm^+^	n.a.	n.a.	n.a.	n.a.	n.a.	n.a.	4
PO_4_^3−^	5	n.a.	5	15	n.a.	n.a.	n.a.
Water	n.a.	57	152	n.a.	47	216	77
Average *B* factor (Å^2^)							
Protein	59.3	65.6	34.4	68.9	47.7	27.7	47.4
Nucleotide	n.a.	57.5	24.6	59.2	36.9	19.4	35.5
Mg^2+^	n.a.	n.a.	n.a.	n.a.	n.a.	21.1	35.0
Gdm^+^	n.a.	n.a.	n.a.	n.a.	n.a.	n.a.	43.9
PO_4_^3−^	40.5	n.a.	49.5	78.6	n.a.	n.a.	n.a.
Water	n.a.	59.4	39.9	n.a.	27.2	26.1	31.1
R.m.s. deviation from ideal values¶							
Bond lengths (Å)	0.006	0.006	0.007	0.006	0.009	0.009	0.010
Bond angles (°)	1.00	0.92	1.23	0.98	1.19	1.24	1.26
Structure validation by *MolProbity* ††							
Ramachandran favoured (%)	95.2	96.8	98.4	96.5	98.1	99.7	99.1
Ramachandran disallowed (%)	0.3	0.0	0.0	0.0	0.0	0.0	0.0
All-atom clash score/percentile	3.91/100th	0.39/100th	0.19/100th	3.0/100th	3.84/99th	2.53/99th	5.17/97th
Poor rotamers (%)	5.6	3.3	1.84	1.84	2.21	3.18	2.94
PDB code	4lj4	4lj5	4lj6	4lj7	4lj8	4lj9	4lja

† *R*_merge_ = 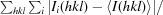 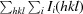, where *I*_*i*_(*hkl*) is the intensity of the *i*th observation of reflection *hkl* and 〈*I*(*hkl*)〉 is the mean.

‡ *R*_work_ = 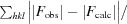 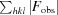.

§ *R*_free_ is calculated like *R*_work_, using 5% of the data that were excluded from refinement.

¶ Ideal values according to Engh & Huber (1991 ▶).

†† Structure validation by *MolProbity* (Chen *et al.*, 2010 ▶).

**Table 2:** ATPase activity and nucleotide-binding parameters of ClpB NBD2 variants

ClpB NBD2 variant	ATPase turnover (min^−1^)	Nucleotide binding			
		Nucleotide	*k*_on_ (µ*M* ^−1^ s^−1^)	*k*_off_ (s^−1^)	*K*_d_ (µ*M*)
Wild-type NBD2†	8.6 ± 2.0	MANT-dADP	4.2 ± 0.02	0.015 ± 6 × 10^−5^	0.0035 ± 2 × 10^−5^
		MANT-dATP	3.9 ± 0.01	0.53 ± 2 × 10^−3^	0.14 ± 7 × 10^−4^
		ADP	2.3 ± 0.04	1.2 ± 0.02	0.51 ± 0.01
		ATP	0.36 ± 0.01	35 ± 0.5	97 ± 2
NBD2 N709A sensor 1 mutant	0.3 ± 0.01	MANT-dADP	5.1 ± 0.02	0.012 ± 1 × 10^−4^	0.0024 ± 3 × 10^−5^
		MANT-dATP	4.7 ± 0.02	0.57 ± 2 × 10^−3^	0.12 ± 8 × 10^−4^
		ADP	2.4 ± 0.03	1.0 ± 0.01	0.43 ± 8 × 10^−3^
		ATP	0.41 ± 0.01	30 ± 0.5	74 ± 3
NBD2 R806A sensor 2 mutant	0.007 ± 0.003	MANT-dADP	2.9 ± 0.01	0.080 ± 3 × 10^−4^	0.027 ± 1 × 10^−4^
		MANT-dATP	2.4 ± 0.01	0.71 ± 4 × 10^−3^	0.29 ± 2 × 10^−3^
		ADP	1.1 ± 0.01	4.4 ± 0.05	3.8 ± 0.06
		ATP	0.52 ± 0.02	44 ± 1	84 ± 4
NBD2 R621Q	0.3 ± 0.04	MANT-dADP	3.0 ± 0.01	0.020 ± 2 × 10^−4^	0.0068 ± 8 × 10^−5^
		MANT-dATP	2.7 ± 0.01	0.29 ± 1 × 10^−3^	0.11 ± 7 × 10^−4^
		ADP	0.94 ± 0.01	1.4 ± 0.01	1.5 ± 0.02
		ATP	0.40 ± 0.01	22 ± 0.4	55 ± 2

† As published in Werbeck *et al.* (2009 ▶).
